# Waist-to-hip ratio, body-mass index, age and number of children in seven traditional societies

**DOI:** 10.1038/s41598-017-01916-9

**Published:** 2017-05-09

**Authors:** M. Butovskaya, A. Sorokowska, M. Karwowski, A. Sabiniewicz, J. Fedenok, D. Dronova, M. Negasheva, E. Selivanova, P. Sorokowski

**Affiliations:** 1 Institute of Ethnology and Anthropology, Russian Academy of Sciences, Moscow, Russia; 2 Russian State University for Humanities, Moscow, Russia; 3 Institute of Psychology, University of Wroclaw, Wroclaw, Poland; 4 Department of Psychotherapy and Psychosomatic Medicine, TU Dresden, Germany; 5 Creative Education Lab, Academy of Special Education, Warsaw, Poland; 6 Moscow State University, Moscow, Russia

## Abstract

It has been suggested that the preference for low WHRs evolved because low WHR provided a cue to female reproductive status and health, and therefore to her reproductive value. The present study aimed to test whether WHR might indeed be a reliable cue to female reproductive history (with lower WHRs indicating lower number of children). Previous studies showed such a relationship for modern and industrialized populations, but it has not been investigated in natural fertility, indigenous, more energy constrained populations facing greater trade-offs in energy allocation than do modern societies. Our sample comprised 925 women aged 13 to 95 years from seven non-industrial societies including tribes from Sub-Saharan Africa (Hadza, Datoga, and Isanzu), Western Siberia (Ob Ugric people: Khanty and Mansi), South America (Tsimane) and South Asia (Minahasans and Sangirese). We demonstrated a culturally stable, significant relationship between number of children and WHR among women, controlling for BMI and age. Based on these data, we suggest that WHR is a reliable cue to female reproductive history, and we discuss our results in the context of previous studies indicating usefulness of WHR as an indicator of health and fertility.

## Introduction

A woman’s WHR is one of the markers of her physical attractiveness and might play role in mate selection (for a review, see: refs 1–3. This parameter reflects the relative distribution of fat in the upper and lower body^1, 4, 5^. The role of WHR in female attractiveness is shown by, e.g., neuropsychological^6^ studies indicating that males show activation in brain reward centers in response to naked female with attractive (~0.7) WHR. Also cross-cultural research conducted among indigenous populations^7–11^ suggest importance of WHR in overall attractiveness. Although the results of these studies are often inconclusive^12, 13^, most data rather indicate that when BMI and average WHR in a given population are controlled, relatively low WHR values are preferred^8, 10, 11^.

It has been suggested that the preference for low WHRs emerged due to certain selective pressures. Singh originally hypothesized that males had an evolved preference for low female WHR, because low WHR provided a cue to female reproductive status and health, and therefore to her reproductive value^1, 5^. Indeed, studies conducted before and after this hypothesis indicate that WHR as a marker of fat distribution predicts several health disorders, such as cardiovascular diseases, adult-onset diabetes, elevated plasma lipids, hypertension, cancer (endometrial, ovarian and breast), gall bladder disease, depression, high stress level, and overall mortality^1, 14–20^. It needs to be noted, however, that many of these health problems (heart diseases, diabetes) were probably not evolutionarily relevant^2, 3, 21^.

Another selective pressure might result from a strong association between WHR and sex hormones. Women with higher WHRs have higher levels of free testosterone^14, 22^ and lower levels of progesterone^17, 23^. Further, estrogens appear to decrease WHRs directly^24^ and indirectly^25^, leading to sex differences in this parameter^26^. Additionally, women with a relatively low WHR have significantly more ovulatory cycles^27^, as well as more regular cycles^28^ than do women with higher WHRs. Based on this evidence, a lower WHR among women may indicate a higher likelihood of conception^29^.

Additionally, WHR is a clear visual cue to a woman’s reproductive status. Following menopause, women’s WHRs becomes similar to men’s^30^. WHR seems also to be a cue to pregnancy, because pregnant women have much higher WHRs than do non-pregnant women^31^. Hence, individual differences in WHRs can broadly indicate current reproductive status, pregnancy and the probability of childbirth^32^. Men appear to be more sensitive to women’s waist circumferences than hip circumferences^33^, which is particularly important given that waist size is a more reliable reproductive indicator than is hip width^4^.

Taken together, it might be inferred that the preference for low WHR results from usefulness of WHR as an indicator of health and fertility. However, there is an additional (non-mutually exclusive) reason that men evolved to use a WHR as a cue to female reproductive value. As parity has an independent effect on WHR^34–43^, waist-to-hip ratio of a sexually mature woman might be a cue to the number of offspring she has had. The fat that adolescent females deposit on the hips and thighs is less metabolically active than is central fat and it is resistant to weight loss, except during late pregnancy and lactation^24^. Some authors suggested that this fat, rich in long-chain polyunsaturated fatty acids (LPUFAs), is particularly necessary for infant brain growth, and is indeed preferentially mobilized for fetal brain development (21; but see: ref. 44. Importantly, the level of this fat decreases with each pregnancy^36^; with each live birth, hip and thigh circumference decreases by 0.5 cm, while waist circumference increases by the same amount. Consequently, a lower WHR might indicate that a woman has had relatively fewer children.

The present study aimed to test whether WHR might indeed be a reliable cue to female reproductive history (with lower WHRs indicating lower number of children). There exist some studies investigating the associations among fat distribution, reproduction and aging^34–43^. However, to the best of our knowledge, no prior study investigated this issue in traditional societies. Thus, it has yet to be documented across cultures that WHR increases with parity in natural fertility, indigenous, more energy constrained populations facing greater trade-offs in energy allocation than do modern societies. Another important reason for considering such populations is that they better approximate reproductive and infectious disease conditions under which the adaptations in question arose. Finally, as distribution of fat might greatly vary between populations^45, 46^, these data from indigenous populations enable testing the universality of results obtained by Lassek and Gaulin or Wells and colleagues^21, 36, 41, 42^. Hence, in the present study we investigated the relationship between WHRs and number of children in traditional populations of women from around the world.

## Material and Methods

### Ethics statement

The study was conducted according to the principles expressed in the Declaration of Helsinki. The study protocol and consent procedure received ethical approval from the Institutional Review Board (IRB) of the University of Wroclaw (Wroclaw, Poland) and Moscow State University (Moscow, Russia). Additionally, we received ethical approval from Tanzania Commission for Science and Technology (COSTECH) and we used archived data collected in the past by Tsimane Amazonian Panel Study^47, 48^ with acceptance of Great Tsimane’ Council (the governing body of the Tsimane’). Our methods were carried out in accordance with the approved guidelines. All subjects gave their informed written consent prior to participation. This was done in a form of their signature if they were able to write, or as a print of their finger. It was deemed appropriate given the low literacy rates among traditional societies and this procedure was specifically approved by the Ethical Boards.

### Participants

The present study was a cross-sectional research conducted in seven traditional societies: Hadza (*n* = 180, age 13–95: *M* = 35.43, *SD* = 16.36, 28 nulliparous women), Datoga (*n* = 200, age 15–88: *M* = 35.38, *SD* = 15.81, 26 nulliparous women), Isanzu (*n* = 51, age 20–65: *M* = 39.80, *SD* = 11.78, 1 nulliparous woman), Ob Ugric people (*n* = 220, age 13–71: *M* = 26.19, *SD* = 12.62, 145 nulliparous women), Minahasans, Sangirese (*n* = 29, age 18–70: *M* = 35.38, *SD* = 15.12, 9 nulliparous women), and Tsimane’ (*n* = 245, age 15–88, *M* = 37.11, *SD* = 18.48, 30 nulliparous women). The age was mostly self-reported and when the participants did not know it, the researchers helped them estimate it based on various events from the past. The data on age of the Tsimane were taken from TAPS database^47, 48^. Below, we present a short description of each society.

### Hadza

The Hadza are a hunter-gatherer society living in Tanzania, Africa. They number approximately 1000–1500 individuals and live in mobile camps, each comprising 30 people on average. This society has been extensively described in the literature (see e.g., refs 49–51. Women typically marry between the ages of 17 and 18 years, whereas men marry around the age of 20. Marriages are typically not arranged, and women typically choose their partners. Because divorce is common, serial monogamy is the best way to characterize the mating system^52^. Although approximately 4% of men have two wives, polygynous marriages in Hadza are not stable, and in most cases may be viewed as a transitory state^53^.

### Datoga

Datoga are Nilotic people who are known in Tanzania under several different names (Datoga, Tatoga, Barabaig, and Mang’ati). They are concentrated mainly in the Arusha, Dodoma, Singida, and Shinyanga regions. The population is estimated to be about 87,978^54^. The Datoga are semi-nomadic pastoralists^55^, polygynous and patrilocal. Eighty five percent of women are in polygynous marriages^56, 57^. Marriage is traditionally arranged by parents, but men frequently arrange their second and later marriages according to personal preferences^56^.

### Isanzu

Isanzu (Ihanzu) are traditional Buntu-speaking agro-pastoralists, living in the Singida region of North-Central Tanzania. Their population size is about 30,000. The Isanzu are settled in 18 villages in the Mkalama District^58, 59^. The Isanzu are divided into 12 exogamous non-localized matri-clans. They are traditionally polygynous. Although in the 1960s’ 25% of men had two or more wives, this practice has become less common, and at present only 7% of married men have more than one wife^60^. Gender relations are asymmetrical, with women being dependent on men for resources throughout their lives, and usually without social power outside of the household^60^.

### Tsimane’

The Tsimane’ are a native Amazonian society of farmer-foragers. Their population of around 8,000 is distributed throughout approximately 100 villages, most of which are located in the area of Beni in northern Bolivia. Tsimane’ are the native Amazonian group, but their level of integration to the Bolivian economy, culture and lifestyle varies a lot between settlements (e. g., ref. 61. This tribe has been extensively described in the literature (e.g., refs 62–64. Similar to other native Amazonian societies, the Tsimane’ still practice cross-cousin marriage. Traditionally, marriage is arranged by parents^65^.

### Ob Ugric (Khanty and Mansi)

The Ob-Ugric people (Khanty and Mansi) are settled on the territory of Russia in Western Siberia and occupy the basins of the Ob and the Irtysh rivers, including their tributaries. According to a census conducted in 2010, the Ob-Ugric people number just over 43 thousand (Khanty–31,000 and Mansi–12,000). Many of them are still practicing traditional occupations, such as fishing, hunting, and reindeer herding, and gathering^66^. Despite the fact that currently most of the Ob-Ugric people live in villages, they still practice nomadic reindeer herding. They are patrilineal; marriages are patrilocal and basically monogamous^67^. Divorces are mainly initiated by women^67^.

### Indonesians (Minahasans and Sangirese)

The Minahasans are inhabitants of Sulawesi. They are a group with the oldest democracy and federal nation among the other Indonesian and Asian tribes due to their old tribal united government^68^. Ancient Minahasa society was both competitive and egalitarian. Minahasa culture does not show any particular discrimination against women^68^. Social status is mainly dependent on personal achievements and the expression of personal virtues^69^. The Minahasans and Sangirese people of Northern Sulawesi are mainly monogamous. They practice agriculture and are known for matri-focal social organization and widespread practice of child adoption and transfer between households^70^.

### Procedure

The studies among the Hadza and Datoga were conducted in the Lake Eyasi region of Tanzania, Africa, between 2012 and 2014, and data on the Isanzu were collected in 2013 in the Mkalama District of the Singida Region, North-Central Tanzania. Data on the Ob Ugric people were collected in 2010 in Khanty-Mansiisk and in the villages of Berezovsky and the Belojarsky Regions of the Khanty-Mansijsky Autonomous District in Russia. Data from the Minahasans and Sangirese were collected in 2014 on the Sulawesi and Sangir Islands in Indonesia. Finally, the data on the Tsimane’ of Bolivia were not collected by the authors of the current paper. They were collected in 2002 to 2006 for a broader Tsimane’ Amazonian Panel Study (TAPS) project in the region of Rio Manique; these data are deposited in a scientific database available upon request from the TAPS members^47, 48^.

According to database provided by TAPS, among Tsimane, circumference of adult was taken at hip in maximal point, and circumference of adult’s waist was measured at belly button (but see also ref. 71. In all other populations, waist circumference was measured with measuring tape horizontally at the narrowest part of the abdominal region. If the narrowest part of the abdominal region was not clearly distinguishable, the waist was measured (still horizontally) midway between the 10th rib and the crest of the pelvic bone^72^. Hips circumference, or “the circumference of the hips through the buttocks”, was measured horizontally in the most protruding points back of the gluteal region, side and front^72^. All people were measured in light clothes, like a clothing called kanga in the case of African groups. When a participant wore heavy clothes (e.g., animal fur), she was asked to remove them before the measurement. Since the data from Tsimane’ and other populations were collected by different research groups, and a slightly different measurement method was applied, descriptive statistics about WHR of Tsimane’ should not be directly compared to the data from other populations.

BMI was measured in a standard way, as weight (measured with scales) divided by height (measured with an anthropometer) squared.

All women who declared being pregnant at the time of investigation were excluded from participation. Similar to previous research^73^, the number of children was self-reported.

## Results

We measured body-mass index (BMI), waist-to-hip ratio (WHR) and number of children among women in each study population. Table 1 presents descriptive statistics and intercorrelations between all variables in the total sample. Table 2 presents means and standard deviations of the estimated WHR in each population depending on the number of children.

**Table 1:** Descriptive Statistics and Intercorrelations between Study Variables.

	Min	Max	M	SD	1	2	3	4
1: Children	0	18	3.51	3.48	1	0.386***	0.121***	0.645
2: WHR	0.609	1.126	0.82	0.08		1	0.280***	0.353***
3: BMI	0.165	44.537	21.00	5.38			1	0.05
4: Age	13	95	33.34	16.29				1

*Note*. *N* = 925. Children = the number of children, WHR = waist-to-hip-ratio, BMI = body-mass index.

***p < 0.001.

**Table 2:** The WHRs of women with different number of children across ethnicities.

Children	Hadza		Datoga		Isanzu		Ob. Urgic People		Tsimane		Indonesians from Sulawesi (Sangirese. Minahasans)		Total	
	*M* (*SD*)	*N*	*M* (*SD*)	*N*	*M* (*SD*)	*N*	*M* (*SD*)	*N*	*M* (*SD*)	*N*	*M* (*SD*)	*N*	*M* (*SD*)	*N*
0	0.79 (0.04)	28	0.77 (0.05)	26	0.83 (−)	1	0.75 (0.05)	145	0.87 (0.06)	30	0.75 (0.05)	9	0.77 (0.06)	239
1	0.79 (0.05)	16	0.76 (0.06)	23	0.83 (0.04)	5	0.81 (0.07)	24	0.88 (0.07)	29	0.79 (0.05)	6	0.82 (0.08)	103
2	0.8 (0.05)	36	0.75 (0.05)	15	0.82 (0.09)	7	0.85 (0.06)	23	0.88 (0.08)	30	0.81 (0.08)	7	0.83 (0.08)	118
3	0.82 (0.05)	21	0.76 (0.06)	32	0.83 (0.05)	6	0.87 (0.06)	16	0.88 (0.04)	16	0.89 (0.09)	3	0.82 (0.07)	94
4	0.82 (0.05)	17	0.75 (0.06)	17	0.85 (0.05)	5	0.88 (0.07)	10	0.89 (0.05)	18	0.87 (0.02)	2	0.83 (0.08)	69
5	0.83 (0.06)	29	0.78 (0.06)	23	0.92 (0.05)	5	1.05 (−)	1	0.95 (0.05)	13	0.74 (0.01)	2	0.84 (0.09)	73
6	0.82 (0.09)	11	0.77 (0.05)	15	0.82 (0.06)	4	—	—	0.93 (0.06)	18	—	—	0.84 (0.1)	48
7	0.8 (0.07)	8	0.79 (0.06)	16	0.88 (0.06)	6	0.8 (−)	1	0.91 (0.06)	17	—	—	0.85 (0.08)	48
8	0.85 (0.1)	5	0.79 (0.05)	10	0.91 (0.01)	2	—	—	0.92 (0.06)	14	—	—	0.87 (0.09)	31
9	0.89 (−)	1	0.77 (0.07)	9	0.83 (0.04)	5	—	—	0.91 (0.05)	16	—	—	0.86 (0.08)	31
10 or more	0.83 (0.08)	8	0.79 (0.08)	14	0.91 (0.08)	5	—	—	0.94 (0.07)	44	—	—	0.9 (0.09)	71
Total	0.81 (0.06)	180	0.77 (0.06)	200	0.86 (0.07)	51	0.78 (0.07)	220	0.91 (0.07)	245	0.8 (0.07)	29	0.82 (0.09)	925

The number of children was significantly skewed (*M* = 3.51, *SD* = 3.47, *min* = 0, *max* = 18, *skewness* = 1.02), therefore it was log-transformed for further analysis. The transformation yielded a more normal distribution of this variable (*M* = 1.18, *SD* = 0.85, *skewness* = −0.12). The raw and log-transformed variables were highly correlated (*r*[985] = 0.93; *p* < 0.001). For the robustness check, all analyses presented below were also performed with a raw number of children as a predictor.

Across populations, the positive correlation between the log-transformed number of children and WHR was statistically significant and robust (*r* = 0.40, *p* < 0.001). A similar effect was observed when the raw number of children was used (*r* = 0.39, *p* < 0.001). To test whether direction and strength of the effect were similar across populations, we analyzed this relationship with the use of multilevel regressions. As a subsample of Indonesians from Sulawesi was quite small in comparison with other populations, in this study we repeated our multilevel regression analyses without and with Indonesians. As the pattern of results was almost exactly the same, we decided to focus on estimates obtained on the entire sample (including Indonesians). Before analysis, we standardized (*z*-scored) the dependent variable (WHR), the predictor (the log-transformed number of children), and control variables: BMI and age in a way that all of our individual level variables had a mean of 0 and a standard deviation of 1 (see: ref. 74. We tested three models: (1) a random intercept model, allowing for differences in the intercept, but assuming the same slope across populations, (2) a random slope model, allowing for different slopes, but assuming the same intercept across populations, and (3) a random intercept and random slope model, allowing both intercepts and slopes to vary among populations. Although the third model (random intercepts, random slopes) provided a significantly better fit than the first (Δ −2LL = 30.79, Δ*df* = 2, p < 0.001) or second model (Δ −2LL = 309.05, Δ*df* = 2, *p* < 0.001), neither the intercept variance (*s2* = 0.19, *SE* = 0.11, *p* = 0.09), nor the slope variance (*s2* = 0.05, *SE* = 0.03, *p* = 0.13) was statistically significant. Hence, we focused on the more parsimonious, random intercept model (see Table 3).

**Table 3:** Multilevel Regression Model with WHR being predicted by number of children, BMI and Age.

Effects	*B* Coefficient (Standard Error)
Fixed Effects	
Constant	0.06 (0.20)
Children (log)	0.15 (0.04)***
BMI	0.18 (0.035)***
Age	0.16 (0.032)***
Random effects	
Individual level variance	0.51 (0.02)***
Intercept variance	0.23 (0.13)
Model properties	−2LL (6) = 1944.52

Note: *** p < 0.001.

The effect of the number of children on WHR was statistically significant (*B* = 0.154, *SE* = 0.037, *p* < 0.001) and remained significant after we partialled out BMI (*B* = 0.18, *SE* = 0.035, *p* < 0.001) and age (*B* = 0.157, *SE* = 0.032, *p* < 0.001), and controlled for grouping participants into different populations.

**Figure 1:**
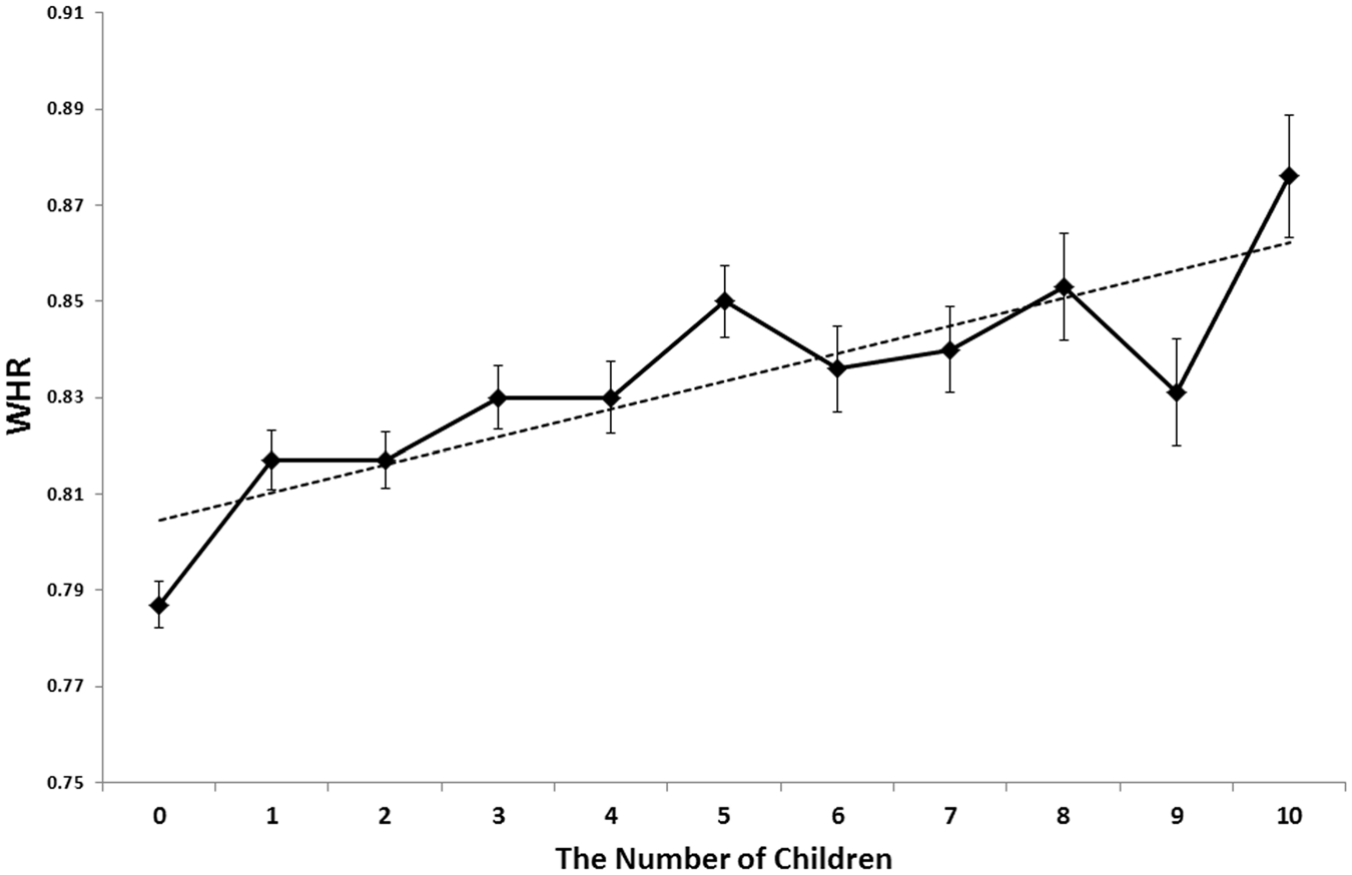
The Relationship between the Number of Children and the WHR (BMI, age and ethnicity are partialled-out).

Importantly, neither the variance of the intercept, nor the slope was significant, indicating that the effect of the number of children on WHR was culturally stable. More specifically, these results showed that the intercept of the WHR was similar across populations, but even more importantly – that slopes in these populations did not differ significantly. This allows us to conclude that this effect is stable across the studied populations. A supplementary multilevel regression analysis with (standardized) raw number of children as predictor, yielded very similar results: the number of children significantly predicted the WHR (*B* = 0.10, *SE* = 0.03, *p* = 0.006), similar to both: the BMI (*B* = 0.19, *SE* = 0.03, *p* < 0.001) and age (*B* = 0.19, *SE* = 0.03, *p* < 0.001). In terms of incremental change of the WHR related to each child, the estimated WHR of females with no children (controlling in the ANOVA for age, BMI and ethnicity) was 0.79 and increased with each child – in the case of females with 10 children, the estimated WHR amounted to 0.88. Even if weak in terms of the effect size, this relationship was statistically significant and linear (Fig. 1).

To perform a robustness check of our results, we provided a two-step procedure. First, we repeated our analyses on a restricted sample, first excluding from the analyses all females who were potentially postmenopausal, i.e., over 50 years old (*n* = 180). All relationships we have previously observed were replicated. Specifically, the effect of the number of children on WHR remained statistically significant (*B* = 0.16; *SE* = 0.043, *p* < 0.001), similarly as the effect of BMI (*B* = 0.17; *SE* = 0.04; *p* < 0.001) and age (*B* = 0.18; *SE* = 0.06; *p* = 0.005). In the last step we additionally excluded all females without children (*n* = 247) from all analyses. Also in this model the number of children significantly predicted WHR (*B* = 0.22, *SE* = 0.065; *p* = 0.001), similarly as BMI (*B* = 0.19; *SE* = 0.044; *p* < 0.001), while age did no longer predict WHR (*B* = 0.09; *SE* = 0.07; *p* = 0.195).

## Discussion

The main result of our study is that the number of children significantly and positively predicted women’s WHRs across several non-industrial populations. The observed effect was statistically significant while controlling BMIs and WHRs that differed across participating societies and generally increased with age in each study population. Our findings indicate that the positive WHR-parity relationship is strongly culturally stable - we observed this association in populations from different continents (Africa, Eurasia and South America), and among nomadic hunter-gatherers, pastoralists and farmers alike. In summary, in line with previous studies conducted among developed societies^34–43^, we argue that the positive association between WHR and number of children may be general for humans. Our findings suggest that WHR is a reliable marker not only of a woman’s reproductive potential, but also of the number of offspring she has birthed.

In order to explore the observed WHR-parity association further, we investigated it among women in reproductive age that had at least one child. WHRs of nulliparous and older women may differ from regular WHRs for reasons other than number of births, and this might influence the effect of number of births on WHR. For example, much of the high WHR of 50–70 year old women may be due to factors other than number of offspring, like obesity or health problems, and yet they would have the most offspring and age, and hence bias the results of the analysis. In the context of our data we can state the overall result does not change regardless of inclusion/exclusion of elderly (possibly postmenopausal) and nulliparous women.

The populations participating in our research differed from one another. However, even when the population factor was included in the analysis, the number of children remained a significant predictor of WHR for the total sample (while controlling for the age and BMI of women). Below, we propose several explanations as to why number of births, associated with lower WHR, can be important for female mate value.

First, as discussed in the Introduction, fewer children mean higher level of LPUFAs that support fetal brain development^21^. Thus, lower WHR (associated with a lower number of previous children) means more LPUFAs for a new partner’s subsequent offspring with the woman. Second, following^2, 3, 75^, limited reproductive potential, and limited windows of female fertile ovulatory cycles in natural fertility populations mean that each child born is probably 1 of 7 or less children a man can sire with the woman in total if he mates with her long-term. Hence, it can be predicted that the preference for a low WHR results from male preference for women at peak residual reproductive value, just prior to first probably fertile ovulatory cycle (and with no previous children). Third, in a short term context, the actual number of fertile days in ancestral forager women’s lives was limited by recurrent pregnancy and lactational amenorrhea^76^. Finally, from an evolutionary point of view, a male investing in another man’s children is synonymous to a man supporting his opponent’s reproductive success^77^. As paternity confidence is one of the most important factors impacting men’s investment in children^78–81^, natural selection typically favors males who avoid taking care of unrelated offspring^82–84^. This phenomenon seems to operate in modern human societies, wherein suspected nonpaternity is one of the most common reasons of refusals to pay child support among American men^85^. Thus, preference for a lower initial WHR in a partner would reduce the probability of mating with a woman who is already a mother of other men’s children. Although there are certain adaptations men have for differential parental solicitude (e.g., Hadza behavior toward step and biological children differs)^86^, attraction to nulliparous women showing cues of fertility, and to those with lower numbers of offspring increase net fitness benefits of mating with those women, and men should be attracted to these cues. Low relative WHR is one of them.

The clear association between female age and WHR that we observed in our research might be also very important in the male mate choice. In almost all participating societies, the youngest women had the lowest WHR (Table 1). Human mate selection has been widely investigated over the past several decades (e.g., refs 87–89, and studies show that men maximise their reproductive success by choosing receptive, highly fecund partners who appear to be potentially successful mothers^90^. One problem related to the male strategy is that there are arguably few direct cues to a woman’s fertility^91, 92^. Thus, men may need to be sensitive to certain indirect cues to potential reproductive value, like female age. Our findings might suggest that preferences for women with lower WHRs can be beneficial in small-scales societies also because WHR may be a cue to younger age across such populations (Table 1). On the other hand, it is important to note that the mean WHR within an age cohort can vary significantly across human populations^11^. Thus, age-WHR associations may be reliable mainly at the within-population level.

Overall, the WHR-related issues discussed above (WHR as a marker of fertility, parity history, health, etc.) suggest that preferences for certain values of this body parameter should be culturally universal. Indeed, most studies show preferences for relatively low WHR within respective populations (7–12; but see: refs 13, 93.

It needs to be noted that the current study has some limitations. First, our design was cross-sectional while it would be more accurate to assess the effect each childbirth has on WHR in a longitudinal study. Second, we compared the data collected by several researchers in a few cultures. This is particularly problematic in the case of different method applied to measure WHRs among Tsimane’. Nevertheless, our research comprise data collected among hundreds of women from several traditional societies, which has an undeniable value; collecting such data would be almost impossible in more a complex, longitudinal design involving only one researcher. Finally, the participating societies differed in sample size and number of nulliparous women. For example, large size of 145 nulliparous Ob Ugric women and their low WHR could potentially affect our results. However, as presented in the results section, even after exclusion of all females without children from all analyses the number of children remains a significant predictor of WHR in the whole sample.

In summary, we demonstrated a culturally stable, significant relationship between number of children and WHR among women, controlling for BMI and age. Based on these data, we suggest that WHR, as a reliable cue to female reproductive history, is an independent and highly valid factor directing the selection of men’s sexual preferences towards women with lower WHRs. Along with selection of younger and healthier women, preferences for low WHRs may enable men to mate with women of highest possible reproductive potential. These findings increase our understanding of sexual preferences in traditional, small-scale societies that approximate reproductive and infectious disease conditions under which the evolutionary adaptations arose.
